# Apical Extrusion of Debris during Root Canal Preparation with ProTaper Next, WaveOne Gold and Twisted Files

**DOI:** 10.3390/ma14216254

**Published:** 2021-10-21

**Authors:** Wojciech Eliasz, Beata Czarnecka, Anna Surdacka

**Affiliations:** 1 Department of Conservative Dentistry and Endodontics, Poznan University of Medical Sciences, 61-701 Poznan, Poland; annasurd@ump.edu.pl; 2 Department of Biomaterials and Experimental Dentistry, Poznan University of Medical Sciences, 61-701 Poznan, Poland; czarnecka@ump.edu.pl

**Keywords:** endodontics, endodontic instruments, root canal preparation

## Abstract

(1) Background: Apical extrusion of debris is an example of a complication that may arise during root canal treatment, and it has been proven to be an unavoidable occurrence during endodontic treatment by numerous authors. Even though it may not hinder the long-term outcome of treatment, it may lead directly to increased levels of postoperative pain and, therefore, lower levels of patient acceptance and satisfaction. The aim of the study was to assess the weight of apically extruded debris during root canal preparation with instruments that use different movement kinematics (rotary, reciprocating, and adaptive motion); (2) Methods: The study was performed using the Myers and Montgomery model. Sixty human premolar teeth were inserted into preweighed Eppendorf tubes and randomly classified into three groups. After manual glide-path preparation, teeth in each group were instrumented to working length set 1 mm short of the anatomical apex using the standard sequence provided by the manufacturers (for Group 1: ProTaper Next X1 & X2; for Group 2: WaveOne Gold Primary, for Group 3: Twisted Files SM1-SM3). Root canals were irrigated with 1 mL of 0.9% NaCl solution between each file insertion. The tubes with collected debris were stored in an incubator at 70 °C for 5 days in order to evaporate the liquid component. Measurement of the weight of extruded debris was performed by subtracting the preinstrumentation from the postinstrumentation weight of the tubes. The results were analyzed with Kruskal–Wallis ANOVA, with significance level set at 0.05; (3) Results: The weight of extruded debris was 0.337 mg (SD = 0.148) for Group 1, 0.305 mg (SD = 0.201) for Group 2, and 0.348 mg (SD = 0.135) for Group 3. (4) Conclusions: Engine-driven root canal preparation with the use of instruments ProTaper Next, WaveOne Gold and Twisted Files that use different movement kinematics (rotary, reciprocating, and adaptive motion) was associated with apical extrusion of debris to a similar extent.

## 1. Introduction

Root canal treatment procedures constitute a significant part of dental treatment provided worldwide, and allow patients to retain their natural dentition with a success rate similar to implant placement procedures [1,2]. However, various complications may occur during its all stages, including perforation, change in the original path of the root canal, instrument fracture, all of which may make it impossible for the clinician to achieve the primary aim of treatment, i.e., infection control and disinfection of the root canal space [3,4]. Apical extrusion of debris is another example of such complication, and it has been proven to be an unavoidable occurrence during endodontic treatment by numerous authors since the methodology for measuring the amount of extruded material was introduced by Myers and Montgomery in 1991 [5]. Several research papers, whose aim was to determine how much tissue debris and what irrigant volumes move beyond the apex, have been devoted to the subject; however, their results are not unequivocal. The only conclusion one can draw from analyzing the data is that virtually all preparation methods are associated with this phenomenon, which in turn can trigger an inflammatory reaction both due to the presence of microorganisms per se in the periapical tissues, and due to physio-pathological phenomena, such as increased gene-expression of endogenous substances and transmitters [6,7]. Due to the huge variety of endodontic instruments available commercially nowadays, researchers have been trying to determine what factors may influence the incidence and severity of this phenomenon, including instrument shape, size, propelling mechanism (hand vs. engine driven), metallurgy, and eventually movement kinematics. The rationale behind conducting this research study was that, along with advances in instrument design and manufacture, it seems that relevant data should be gathered for new systems available for purchase [8]. As movement kinematics may play a role in the severity of debris extrusion from the root canal, three, popular groups of instruments used worldwide that use rotary, reciprocal, and adaptive motion, were chosen to the study. Even though each group claims to be safe, according to the manufacturer’s guidelines and information, some differences in technical performance have been observed between files that belong to each of the aforementioned groups. Some studies showed different potential for inducing root canal cracks, others—for root canal transportation. Therefore, due to introduction of ProTaper Next (Dentsply Sirona, Charlotte, NC, USA) rotary system and WaveOne Gold (Dentsply Sirona, Charlotte, NC, USA) reciprocal, and Twisted Files (Kerr Endodontics, Orange, CA, USA), and due to the fact that limited data is available on the topic, and post-operative pain resulting from extrusion of significant amounts of debris can decrease the acceptability of the treatment in patients, we chose to compare the amount of extruded debris after preparation with these three instrument systems and to determine whether the differences are statistically significant. The null hypothesis in the study was that there will be no difference in the amount of apically extruded debris after preparation with these three systems.

## 2. Materials and Methods

Sixty freshly extracted human single-rooted teeth were used for the study. Criteria for including teeth included patient’s age (25–40 years of age), no parafunction reported or observed in clinical examination, and no history of previous trauma or evidence of root cracks. The roots were debrided on the external surface using sterile gauze and saline. Afterwards, straight-line access to the root canals was achieved by preparing a repeatable reference point within the crown. Working length was estimated for each root canal by inserting a size 10 C-Pilot (VDW, Munich, Germany) into the root canal until its tip reached the apical foramen, which was observed under magnification. Then the file was retracted 1 mm. The experimental model for measuring the amount of extruded debris was then prepared according to the methodology described by Myers and Montgomery. Each Eppendorf tube was weighed three times before the experiment (Radwag, Radom, Poland), and the mean value for the weight was then obtained. The tooth was mounted in silicone putty covering the tube opening. A 25-gauge injection needle was placed through the cover in order to equalize the pressure inside and outside of the tube. The tube was then covered with black opaque tape in order to blind the operator as to the amount of extruded debris. Root canals were then prepared by one operator in the following manner: glide-path was obtained in all teeth by preparing them manually with stainless-steel K-files (Poldent, Warsaw, Poland) to size ISO 20 (20%). The teeth were then randomly divided into three groups Random Sequence Generator (Random.org, Dublin, Ireland) software, each group containing 20 teeth. In group 1, root canal preparation was achieved using PTN X1 and X2 files; in group 2, using the WaveOne Gold Primary file; and in group 3, Twisted Files sizes SM1-SM3. Root canals were irrigated with 5 mL of 0.9% NaCl solution between each file insertion (side-vented 30G needles were used for irrigation). The files were used following the manufacturer’s guidelines regarding the speed and torque:

- Group 1: ProTaper Next (PTN)—X-Smart endomotor (Dentsply Sirona, Charlotte, NC, USA), 300 rpm, torque 2.0 Ncm; size X1 and X2
- Group 2: WaveOne Gold (WOG)—X-Smart endomotor (Dentsply Sirona, Charlotte, NC, USA)—WOG reciprocating mode; size: WOG Primary
- Group 3: TF Adaptive (TF)—Elements Motor endomotor (Kerr Endodontics, Orange, CA, USA)—Adaptive Motion program; size SM1 (20; 0.04), SM2 (25; 0.06), SM3 (35; 0.04).

After instrumentation, the model was dismounted and the debris adhering to the outer surface of the root was washed with 0.9% NaCl solution and placed in the tube. All tubes were placed into an incubator and stored there for 5 days (temperature set at 70 degrees Celsius) in order to evaporate the irrigant. Afterwards, all tubes were weighed three times once again using the same equipment and in the same conditions. Mean value for the weight of extruded debris was calculated by subtracting the preoperative weight from the post-operative weight of the tube. 

Statistical analysis was performed using the PAST4.03 software [9]. The results were analyzed with Kruskal–Wallis ANOVA. *p*-value of <0.05 was the significance value set for the statistical tests used in the study.

## 3. Results

**Figure 1:**
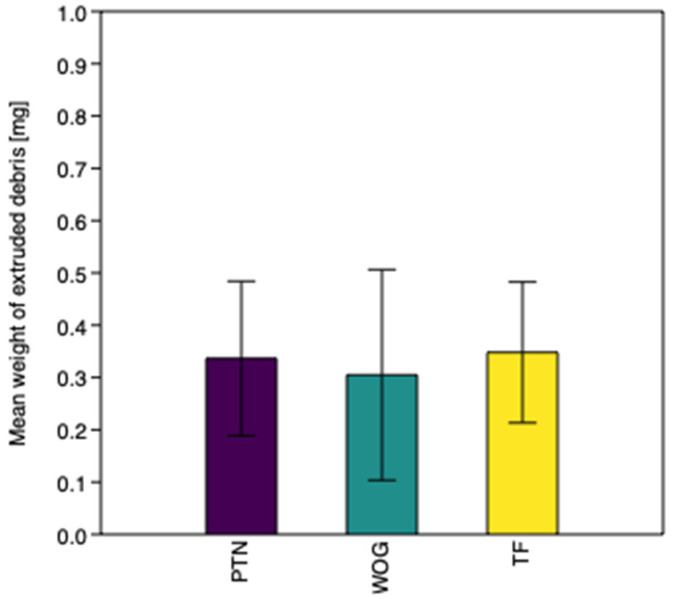
Mean weight of extruded debris for experimental groups.

Apical extrusion of debris was observed in all groups. The weight of extruded debris was 0.337 mg (SD = 0.148) for Group 1, 0.305 mg (SD = 0.201) for Group 2, and 0.348 mg (SD = 0.135) for Group 3. The values are represented graphically in Figure 1. Statistical analysis showed no significant differences between the experimental groups (*p* = 0.224).

## 4. Discussion

This study discusses the phenomenon of apical extrusion of debris between two endodontic instrument systems using different movement kinematics—rotary (ProTaper Next, Dentsply Sirona) and reciprocal (WaveOne Gold, Dentsply Sirona, and adaptive motion (TF Adaptive (TF), Kerr Endodontics, Orange, CA, USA)). The files were chosen due to their similarity in cross-section and size. The methodology that was used for the study has been used for the purpose of measuring the amount of apically extruded debris for several years in various studies published in endodontic journals regarding new preparation systems available for purchase [10,11,12]. This approach is repeatable and effective, and the results from one study can be easily compared with other studies, which provides appropriate possibilities for further meta-analyses between different studies. Nevertheless, there are some limitations to the methodology used—there are no structures that represent periapical bone, operator-related factors (e.g., force of irrigation), or the choice of irrigant (NaCl). First of all, this model may exhibit certain shortcomings, i.e., there is no structure that would represent the periapical bone, which, in physiological environment, constitutes a point of resistance [8]. To overcome this issue, two solutions have been proposed—the use of an artificial substance, which could imitate it, or performing the study in cadavers or in patients. The first group included, e.g., floral foam, as its porous structure is believed to be similar to that of the bone [13,14,15]. This approach has not gained popularity, though, because the irrigant and debris are trapped in the porosities and may not be extracted, which makes it impossible to see the whole picture. Additionally, other studies have been performed in patients using, for example, the addition of contrast medium to the irrigant, or the measurement of the concentration of inflammatory markers in periapical fluid [6,16]. Nevertheless, all contrast medium types may induce a potentially allergic reaction in some patients, and reproducibility of periapical radiography and interrater agreement in assessment of the quality and quantity of the bone is not high [17,18]. Theoretically, the use of cone beam computed tomography could prove helpful, but this is associated with unnecessary exposure to radiation [19]. Therefore, the methodology used in our study remains the most practical and reproducible option for such measurements, and also enables comparison with other studies. Secondly, the fact that a single operator can perform the study constitutes both a strong point, and a limitation. All results can be compared in an easy and reproducible way, but on the other hand, the force of extruding the irrigant through the needle could be different for different operators. This, however, has not been discussed as being an important factor in study methodologies published by other authors [8]. To overcome the possibility of data miscalculation, the specimens were weighed three times in a random manner in order to minimize any potential errors. Additionally, the choice of irrigant can be regarded as a limitation. Even though sodium hypochlorite is the most commonly used irrigant, we decided that it would be best to use a fluid that is seen as a neutral irrigant, in order to focus on the physics of instrument movement. Other fluids (EDTA, citric acid, chlorhexidine, or mixed irrigants—MTAD, QMix) are available for use and are indicated in certain circumstances [20], but to include them in the study, it would be necessary to change the methodology and statistics, as all properties of the irrigants would have to be evaluated in detail beforehand.

There is another issue to be taken into account when discussing the methodology of the study. Sodium hypochlorite is the most commonly used irrigant in contemporary endodontics, and it has been used in some studies. However, this solution is not inert when in contact with organic tissues. Apart from disinfection, it dissolves organic debris and dentin, and it may also crystallize [21]. Therefore, as the aim of the study was to investigate the physical aspects of irrigation, a biologically inert solution was used in order to see the clear picture, and, as new irrigants are under development, the results can be translated more easily to new solutions that may become popular in future [22]. Additionally, several aids for irrigation have been developed, such as photoactivation or sonic activation, and studies regarding the occurrence of this complication constitute an interesting point for further research studies. Furthermore, as the need and acceptance for endodontic revascularization procedure increases, during which irrigation plays an important role, the influence of extruded liquid on the cells that are said to be responsible for the re-growth of tissue into the root canal constitutes an interesting direction for further research [23,24].

With respect to the results of our study, they are similar to observations made by other teams, such as Boijink et al. [25], Capar et al. [26], Kirchhof et al. [27], Yilmaz and Ozyurek [28]. All these studies show that apical extrusion of debris is an inherent complication during endodontic treatment, but they compared similar files in different combinations. A meta-analysis of the results [29] concluded that stainless steel (SS) hand instruments tend to extrude more debris, but the differences between engine-driven preparations are similar, which is also shown in our study. Even though there may be several explanations as to why it happens, an interesting point can be made when comparing our study with the study by Kirchhof et al., as they used similar instruments, only a different version of the WaveOne file [27]. The fact that both WO in their study and WOG in our study were shown to extrude the least amounts of debris may indicate that cross-section of the instrument and thermomechanical treatment do not influence it to a significant extent. Therefore, the final outcome may also be influenced by the instrument design. Both ProTaper Next instruments and WaveOne Gold Primary have off-set centers of rotation, and this is believed to cut dentine of the root canal more effectively within its whole diameter. Moreover, this provides more space for the debris to be transported, as has been shown in other research studies that compared the ProTaper Universal system with ProTaper Next [21]. Data regarding apical extrusion of debris with the use of TF remains scarce. Even though the manufacturer claims that the instrument movement within the root canal is gentle for dentin and does not promote extrusion of debris, our study showed results that were similar to observations made by other teams [12,30], who observed higher values for extrusion when adaptive motion was used. Nevertheless, the amounts remain small and statistically insignificant. This may be explained by the design of the instrument, as PTN and WOG have off-set centers of rotation, which allows the dentin debris to move more freely towards the coronal part of the root canal. An interesting observation was made by Liu et al. [31], who noted that the amounts of debris extruded decreased along with an increase in rotational speed—this may constitute an interesting point for further research studies.

As with all endodontic complications, extrusion of infected debris may constitute a clinically significant occurrence, as it may be theoretically related to, for example, post-operative pain or extraradicular infection. However, animal models show that apical inflammation resolves over time even in the presence of infected debris; however, post-operative pain may remain a significant issue, as it may influence further adherence to dental treatment plan [32,33]. Apical extrusion of debris is a phenomenon that is relevant clinically, as it may cause result in post-operative pain and flare-up of an already existing endodontic pathology. According to what is known nowadays, it is not only the quality of the debris that plays significant role (i.e., the virulence of microorganisms and microbial load), but also their quantity, as well as the mere presence of a “foreign body” over the apex [34]. This stimulates several pathways of inflammatory reaction by stimulating, for example, the expression of substance P and Calcitonin gene-related peptide, which, in turn, are related to the development of neurogenic inflammation [6,35]. This can decrease the acceptability of the treatment between patients and, in the most severe cases, may even lead to treatment failure. The differences in pain levels have been examined e.g., by Gambarini et al. [36], Hou et al. [37] or Nekoofar et al. [38], who concluded that more patients in whom reciprocal preparation was used experienced post-operative pain. However, this phenomenon cannot be excluded completely; therefore, other treatment possibilities, e.g., pharmacology or cryotherapy may be useful [39,40]. Furthermore, the alloy used for instrument manufacture may play some role. ProTaper Next, WaveOne Gold, and Twisted Files are made of heat-treated alloy, which makes them less stiff and allows for easier adaptation to the original shape of the root canal [41].

## 5. Conclusions

Root canal preparation using rotary ProTaper Next instruments, reciprocal WaveOne Gold instruments, and Twisted Files using adaptive motion are associated with apical extrusion of debris to a similar extent. Due to the standardization of performance, the null hypothesis, i.e., that there is no difference between these systems, was accepted.

## Data Availability

The datasets generated during and/or analysed during the current study are available from the corresponding author on reasonable request.
